# Significance of Enrupatinib, a CSF1R inhibitor, on Decreasing Neuroinflammation and Maintaining Neuronal Health in Mice Models of Alzheimer's Disease

**DOI:** 10.1002/alz70859_102542

**Published:** 2025-12-25

**Authors:** Wahyu Dewi Tamayanti, Agnes Dwi Ariyanti, Haw Yuan Cheng, Chia Wei Huang, Chi Yun Pai, Hung‐Kai Chen, Jin‐Wu Tsai

**Affiliations:** ^1^ Institute of Brain Science, Schools of Medicine, National Yang‐Ming Chiao Tung University, Taipei, Taiwan, Taipei City Taiwan; ^2^ Taiwan International Graduate Program in Molecular Medicine, National Yang Ming Chiao Tung University, and Academia Sinica, Taipei Taiwan; ^3^ Elixiron Immunotherapeutics, Taipei City Taiwan; ^4^ Elixiron Immunotherapeutics, Taipei Taiwan; ^5^ Institute of Brain Science, Schools of Medicine, National Yang‐Ming Chiao Tung University, Taipei, Taiwan, Taipei Taiwan

## Abstract

**Background:**

A strong innate immune response, including microglial activation and high levels of inflammatory cytokines, is prevalent in Alzheimer's disease (AD), contributing to neuroinflammation, neuronal damage, and cognitive decline. Modulating microglial activity is therefore a promising therapeutic strategy. We explored enrupatinib, a brain‐penetrant CSF1R inhibitor, as a potential disease‐modifying agent using AD mouse models 5xFAD and J20. This study focused on the effects of enrupatinib on neurons and microglia.

**Method:**

We performed in utero electroporation on layer V neurons of embryonic‐day‐12.5 5xFAD mice, tagging them with GFP. Postnatally, we administered enrupatinib at doses of 300 mg/kg. Behavioral assessments, including the Novel Object Recognition (NOR) test and Y‐maze, were conducted to evaluate cognitive function. Additionally, we examined microglia, Aβ plaques, and GFP‐tagged neurons in brain sections using microscopy. To analyze microglial biomarkers and neuroinflammation‐related pathways, we employed immunofluorescence, quantitative PCR (qPCR), western blotting, and RNA sequencing.

**Result:**

Enrupatinib therapy significantly lowered neuroinflammatory markers, microglial proximal to plaque density, and but preserving the microglia distal to plaque. It also diminished the expression of disease‐associated microglia (DAM)‐related genes, demonstrated a tendency for lower amyloid plaque deposition, and enhanced cognitive function in the NOR and Y‐maze tests. The injection of enrupatinib significantly minimized fragmented neuronal cells. After treatment, RNA sequencing showed that pathways related to inflammation were lowered in both the 5xFAD and J20 models.

**Conclusion:**

These findings underscore the pharmacological potential of enrupatinib in mitigating Alzheimer's disease‐related pathologies. By reducing neuroinflammation and preserving neuronal integrity, enrupatinib offers a promising therapeutic avenue for Alzheimer's disease.

